# The Use of Local Ingredients in Shaping Tourist Experience: The Case of *Allium ursinum* and Revisit Intention in Rural Destinations of Serbia

**DOI:** 10.3390/foods14091527

**Published:** 2025-04-27

**Authors:** Tamara Gajić, Sonja P. Veljović, Marko D. Petrović, Ivana Blešić, Milan M. Radovanović, Slavica Malinović Milićević, Ana Milanović Pešić, Yerlan Issakov, Dariga M. Khamitova

**Affiliations:** 1 Geographical Institute “Jovan Cvijić”, Serbian Academy of Sciences and Arts, 11000 Belgrade, Serbia; m.petrovic@gi.sanu.ac.rs (M.D.P.); m.radovanovic@gi.sanu.ac.rs (M.M.R.); s.malinovic-milicevic@gi.sanu.ac.rs (S.M.M.);; 2 Swiss School of Business and Management, Geneva Business Center, Avenue des Morgines 12, 1213 Genève, Switzerland; 3 Faculty of Hotel Management and Tourism, University of Kragujevac, 36210 Vrnjačka Banja, Serbia; 4 Department of Regional Economics and Geography, Faculty of Economics, Peoples’ Friendship University of Russia (RUDN University), 117198 Moscow, Russia; 5 Department for Geography, Tourism and Hotel Management, Faculty of Sciences, University of Novi Sad, 21000 Novi Sad, Serbia; ivana.blesic@gmail.com; 6 Institute of Environmental Engineering, Peoples’ Friendship, University of Russia (RUDN University), 6 Miklukho Maklaya St., 117198 Moscow, Russia; 7 Department of Geography and Ecology, Faculty of Natural Sciences and Geography, Abai Kazakh National Pedagogical University, Almaty 050000, Kazakhstan; 8 Department of Art and Art Management, Kazakh National Academy of Choreography, Uly Dala Str. 43/1, Astana 010000, Kazakhstan; dariga1979@mail.ru

**Keywords:** affective response, attachment to food, authenticity, cognitive response, emotional involvement, flow state, gastronomic tourism, sensory perception

## Abstract

This study explores the role of *Allium ursinum* L. in shaping authentic gastronomic experiences and its influence on tourists’ perceptions and revisit intentions in rural destinations. *Allium ursinum* was selected due to its cultural symbolism, seasonal availability, and traditional culinary use, particularly in Serbia. The study applies the SOR (Stimulus–Organism–Response) model to investigate how cognitive and affective responses elicited by interactions with this plant shape tourist behavior. A structured questionnaire was administered to 336 tourists who had consumed food containing *Allium ursinum* in ten rural destinations across the Republic of Serbia. The findings emphasize the importance of cognitive responses, such as authenticity perception, knowledge acquisition, and cultural understanding, which in turn influence affective responses, including enjoyment, emotional involvement, and flow state. While positive emotions enhance attachment to local food and revisit intention, the subjective nature of these assessments calls for caution when generalizing. The study contributes to the literature by identifying sensory and emotional mechanisms that link local food ingredients with tourist loyalty. This is the first study to empirically test the SOR model using a local plant ingredient in a rural tourism context.

## 1. Introduction

**Figure 1:**
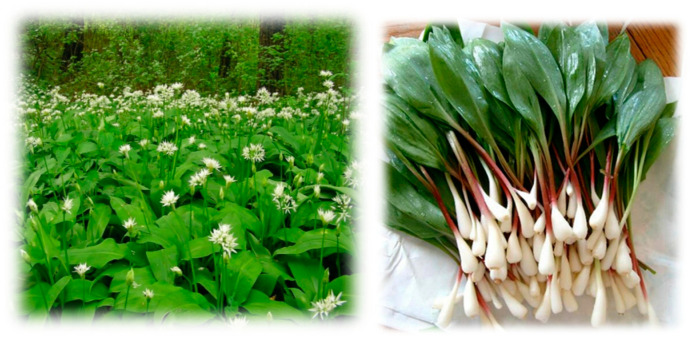
Leaves and bulbs of wild garlic. Source: www.bing.com.

Rural tourism, as a growing segment of the global tourism industry, emphasizes authentic experiences that rely on specific natural resources, cultural heritage, and local gastronomy [1]. Tourists’ desire for authentic experiences often leads them to rural areas where they can engage with traditional ways of life and unique local products [2]. The role of local ingredients in shaping the tourist experience is particularly significant, including plants used in traditional cuisine and folk medicine (Figure 1) [3]. Although research often focuses on the importance of local food and specific ingredients in creating unique tourist experiences, less attention has been paid to studying the impact of individual plants with distinctive characteristics on visitors’ perceptions and satisfaction [4]. One such ingredient is *Allium ursinum*, which is traditionally used in Serbia and across the Balkans for its nutritional value, distinctive flavor, and health benefits [5]. Research on *Allium ursinum* has primarily focused on its biochemical composition, while its potential for enhancing authentic tourist experiences has been overlooked, despite the growing interest in unique gastronomic experiences within rural destinations [6,7,8,9,10].

Analyses of the nutritional and chemical properties of *Allium ursinum* have demonstrated high levels of minerals such as iron, phosphorus, sodium, and copper, as well as vitamins A and C, dietary fibers, and phenolic compounds [11,12,13,14,15]. Particularly notable are sulfur compounds, which are essential for culinary use due to their intense flavor and positive health effects [16,17,18,19,20]. Additionally, *Allium ursinum* has been identified as a natural preservative with strong bactericidal properties, which further enhances its value in culinary practices [21,22,23]. Lachowicz et al. [24] emphasized that the phenolic content and antioxidant capacity of wild garlic contribute to its aroma and nutritional value, making it distinct compared to other plants. Similarly, Voća et al. [25] highlighted that the antioxidant properties of wild garlic contribute to a rich flavor profile, making it an attractive ingredient in regional dishes. However, the ways in which these attributes shape tourists’ cognitive and affective responses, or influence their perceptions of destination authenticity, remain unexplored.

The use of indigenous plants in culinary practices represents a significant element in creating authentic tourist experiences, particularly in rural areas where local resources and cultural heritage play a pivotal role in shaping gastronomic offerings [26,27,28]. Authenticity of experience, which relies on specific local ingredients, is increasingly important in differentiating tourism offerings, enhancing the attractiveness of rural destinations, and promoting their sustainability [29,30]. Vafadari et al. [4] emphasized the importance of resilience in local food systems for creating authentic experiences, while Cooke [7] highlighted that specific food ingredients are essential for shaping the tourist experience among visitors seeking unique gastronomic offers. Similarly, González and Lorenzo-Cuyás [31] applied the “social heroes” approach to promote local products, underscoring their significance for sustainable development and the preservation of local culture. Farkić, Filep, and Taylor [32] suggested that guided adventures based on local food can enhance tourists’ well-being, emphasizing the role of authenticity in forming positive tourism experiences.

Although it is assumed that authentic gastronomic experiences can stimulate positive emotional reactions and increase revisit intention, the existing body of research on this topic remains relatively limited, particularly in the context of rural destinations [33,34]. This is especially true for lesser-known plants such as *Allium ursinum* (wild garlic), whose tourism potential remains largely underexplored, especially within less commercialized or peripheral regions [21,35]. Plants such as wild garlic, rosemary, thyme, and basil are commonly used in regional recipes, valued not only for their distinctive flavors but also for their role in preserving and conveying cultural heritage across generations [36,37,38,39,40]. Although many studies have examined the nutritional and medicinal properties of these herbs, their contribution to shaping memorable gastronomic experiences and enhancing the uniqueness of rural tourism offerings has received comparatively less scholarly attention [35,41,42,43,44,45].

### Theoretical Framework and Hypothesis Formulation

The theoretical framework of this study is based on the Stimulus–Organism–Response (SOR) model, originally developed by Mehrabian and Russell [46] and further conceptualized by Jacoby [47]. This model has become one of the most prominent frameworks for analyzing consumer and tourist behavior in various experiential contexts. It provides how external environmental stimuli (S), such as exposure to *Allium ursinum* in rural gastronomy, evoke internal cognitive and affective responses (O) within individuals, which subsequently lead to specific behavioral intentions (R), including revisit intention and attachment to local food. In this study, the authentic gastronomic experience serves as the stimulus, eliciting both knowledge-based (cognitive) and emotion-based (affective) internal states among tourists. These internal responses are expected to influence behavioral outcomes, particularly loyalty to the destination and the desire to revisit. By applying the SOR model, this research provides a structured and comprehensive understanding of how *Allium ursinum*, as a unique local ingredient, contributes to shaping authentic tourism experiences and behavioral intentions. The following hypotheses are derived in alignment with this theoretical model, and each is supported by relevant literature and empirical findings.

**Figure 2:**
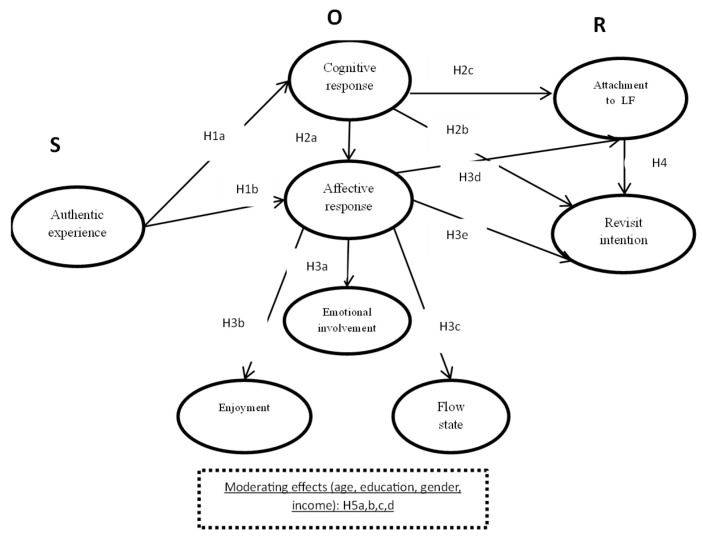
A flow diagram and hypothesis framework using the SOR model.

While previous studies have primarily focused on the nutritional and health characteristics of plants or their commercial use in the food industry, the integration of sensory properties and perceptions of authenticity within a tourism context remains underexplored [48,49]. The identified gap in research addressing the relationship between specific sensory attributes of plants and tourists’ perceptions of authenticity highlights a significant research gap. Based on the identified research gaps, a theoretical framework has been developed that includes the following key constructs: authentic experience, cognitive response, affective response, emotional involvement, enjoyment, flow state, attachment to local food, and revisit intention (Figure 2).

Stimulus (S): Authentic experience

The authentic experience in this study refers to tourists’ interactions with the specific local ingredient *Allium ursinum*, which is used in the traditional cuisine of rural destinations. Previous research suggests that authentic experiences play a crucial role in differentiating tourism offerings, particularly in rural areas where the use of local ingredients can provide unique experiences [26,50]. Authors such as Dimitrijević et al. [40] and Blešić et al. [34] emphasized that authentic experiences positively influence tourists’ cognitive responses, including perceptions of authenticity, knowledge acquisition, and understanding of the cultural context. Lin and Hsu [51] added that authentic experiences can enhance support for sustainable development, while Vafadari et al. [4] highlighted the importance of resilience in local food systems for creating authentic experiences. Authentic gastronomic experiences play a central role in shaping tourists’ perceptions and emotional engagement. Cooke [7] emphasized the significance of specific food ingredients in constructing meaningful tourism experiences, while González and Lorenzo-Cuyás [31] highlighted the role of local products in promoting authenticity and supporting sustainable destination development. In line with the Stimulus–Organism–Response (SOR) framework, *Allium ursinum* is conceptualized as a stimulus that initiates internal cognitive and affective reactions. The authentic experience of interacting with this culturally embedded and seasonal ingredient is expected to enhance tourists’ understanding and emotional connection to the place. Based on these assumptions, the following hypotheses are proposed:

**H1a.**  *Authentic experience with Allium ursinum positively influences tourists’ cognitive response (e.g., perceived authenticity, knowledge acquisition).*

**H1b.**  *Authentic experience with Allium ursinum positively influences tourists’ affective response (e.g., emotional involvement, enjoyment).*

Organism (O): Cognitive and affective responses

Cognitive responses relate to perceptions of authenticity, knowledge acquisition, and understanding of the cultural context [52,53,54]. Lin and Hsu [51] emphasized that authentic experiences not only enhance perceptions of authenticity but also encourage support for sustainable development, which is particularly relevant for rural destinations that rely on local ingredients.

Affective responses include tourists’ emotional reactions, such as enjoyment, emotional involvement, and flow state. The flow state refers to a mental condition characterized by deep immersion, concentration, and intrinsic enjoyment, often experienced during meaningful and engaging activities. In the context of gastronomic tourism, it represents a moment when tourists become fully absorbed in the experience, losing awareness of time and external distractions. Jokom et al. [55] highlighted that authentic culinary experiences positively influence tourists’ emotional reactions, including the emergence of flow, which contributes to greater destination loyalty. Similarly, Dai et al. [56] stressed that positive affective responses, such as emotional involvement and enjoyment, can strengthen attachment to local food and enhance revisit intentions.

According to the SOR model, cognitive evaluations typically precede and shape affective responses. In the context of gastronomic tourism, when tourists perceive an experience as authentic and gain knowledge related to local food and culture, these cognitive responses can stimulate positive emotional states such as enjoyment, emotional involvement, and immersion. Based on previous empirical findings, the following hypotheses are proposed to capture the directional relationships between cognitive and affective components, and between affective components and behavioral intentions:

**H2a.**  *Perceived authenticity and knowledge acquisition (cognitive response) positively influence the sense of emotional connection (affective response).*

**H2b.**  *Cognitive response positively influences tourists’ emotional involvement with the experience.*

**H2c.**  *Cognitive response positively influences the degree of enjoyment experienced by tourists.*

**H2d.**  *Cognitive response positively influences the emergence of a flow state during the gastronomic experience.*

**H3a.**  *Affective response positively influences tourists’ emotional involvement.*

**H3b.**  *Affective response positively influences enjoyment.*

**H3c.**  *Affective response positively influences the experience of flow state.*

**H3d.**  *Affective response positively influences tourists’ behavioral intentions, including attachment to local food and revisit intention.*

**H3e.**  *Affective response positively influences tourists’ revisit intention.*

Response (R): Behavioral intentions

In this study, behavioral intentions refer to tourists’ willingness to revisit a destination and exhibit loyalty toward it. According to the Stimulus–Organism–Response model developed by Mehrabian and Russell [46], positive affective reactions can lead to favorable behavioral outcomes, such as enhanced satisfaction and destination loyalty [27,41,57,58]. Sobolewska et al. [59] and Vukolić et al. [37] highlighted that a favorable attitude toward local food, especially when shaped through authentic gastronomic experiences, increases the likelihood of return visits. Jokom et al. [55] emphasized that emotional involvement and perceived authenticity significantly strengthen tourists’ attachment to local food, which, in turn, fosters loyalty to the destination. Similarly, Dai et al. [56] and Xu et al. [60] confirmed that emotional responses can deepen food-related attachment, directly influencing tourists’ behavioral intentions. Based on these insights, the following hypothesis is proposed:

**H4.**  *Attachment to local food positively influences tourists’ behavioral intentions, including revisit intention and destination loyalty.*

In addition to direct effects, this study examines the potential moderating role of demographic factors, age, education, gender, and income on the relationship between affective responses and behavioral intentions. Prior research by Mahmood et al. [50] and Shahrajabian et al. [10] suggested that different visitor segments exhibit varying perceptions of authenticity and levels of satisfaction, which may in turn shape their emotional engagement and loyalty-related behaviors. For instance, older tourists are often more receptive to culturally immersive and authentic experiences, showing higher levels of emotional involvement and a greater likelihood of repeat visits [56,58,61]. These demographic characteristics can thus influence the strength or direction of the relationship between affective responses and revisit intention. Based on this rationale, the following hypotheses are proposed:

**H5a.**  *Age moderates the relationship between affective response and revisit intention, such that the effect is stronger among older tourists.*

**H5b.**  *Education moderates the relationship between affective response and revisit intention, with more educated tourists exhibiting a stronger association.*

**H5c.**  *Gender moderates the relationship between affective response and revisit intention.*

**H5d.**  *Income moderates the relationship between affective response and revisit intention.*

The aim of this study is to examine how the specific local ingredient, *Allium ursinum*, contributes to shaping tourists’ perceptions of authenticity and satisfaction in rural destinations. The research problems arise from the underexplored potential of *Allium ursinum* in enhancing tourist experiences and its impact on tourists’ behavioral intentions. Additionally, the study seeks to assess tourists’ cognitive and affective responses to experiences with *Allium ursinum*, as well as their revisit intentions. The application of the SOR model framework allows for an integrated understanding of how a specific local ingredient like *Allium ursinum* acts as a stimulus that evokes certain cognitive and emotional responses in tourists, which subsequently influence their behavioral intentions. Accordingly, this study seeks to answer the following research question:

How does an authentic gastronomic experience involving *Allium ursinum* influence tourists’ internal responses and behavioral intentions in the context of rural tourism?

The broader significance of this study lies in its potential applicability to various cultures and specific ingredients worldwide. In many destinations, certain local ingredients represent key elements for creating authentic gastronomic experiences that enhance the attractiveness of tourism offerings. It is known that ginger in Chinese cuisine, olive oil in Mediterranean countries, and vanilla in the Caribbean represent more than mere ingredients; they are symbols of cultural identity and authenticity that attract tourists through their uniqueness [8,9,10]. Similarly, *Allium ursinum* can play a comparable role in differentiating the tourism offering in Serbia, providing tourists with a unique experience connected to local tradition and culinary heritage. This approach not only contributes to enriching the tourism product but also promotes the cultural authenticity of destinations, which can have positive implications for their competitiveness and sustainability.

While several plant species such as *Taraxacum officinale* and *Sideritis* spp. (mountain tea) are widely recognized and traditionally used in the Balkan region [20], *Allium ursinum* was selected for this study due to its unique combination of factors relevant to rural tourism. Firstly, it is a highly seasonal wild edible plant, harvested in early spring, which increases its perceived authenticity and experiential value among tourists. Secondly, its intense flavor, strong cultural symbolism, and deep-rooted presence in local gastronomy make it particularly suited for studying sensory-driven experiences. Unlike more widely commercialized plants, *Allium ursinum* is typically foraged in forested rural areas, often involving tourist participation in harvesting and preparation processes, which enhances its potential as a stimulus in the context of the SOR model. Thus, its use supports a more immersive and distinctive rural tourism experience, differentiating it from better-known or industrially commodified herbal species. In recent years, *Allium ursinum* has also gained visibility within regional tourism practices and local product innovation. The “Festival of Wild Garlic” in Eastern Serbia, informal local markets in Golija and Rtanj, and seasonal gastronomic events in the Fruška Gora region celebrate the plant’s cultural and culinary relevance. Furthermore, small-scale producers offer value-added products such as *Allium ursinum* pesto, and pickled varieties, reinforcing its role in rural tourism narratives and regional identity-building.

Figure 2 presents the conceptual framework of the study, which is based on the Stimulus–Organism–Response (SOR) model. The model illustrates how the authentic experience with *Allium ursinum* (stimulus) generates cognitive and affective responses (organism), which subsequently influence behavioral intentions such as attachment to local food and revisit intention (response). The framework also includes moderating variables (age, education, gender, and income) affecting the relationship between affective response and revisit intention.

## 2. Materials and Methods

**Figure 3:**
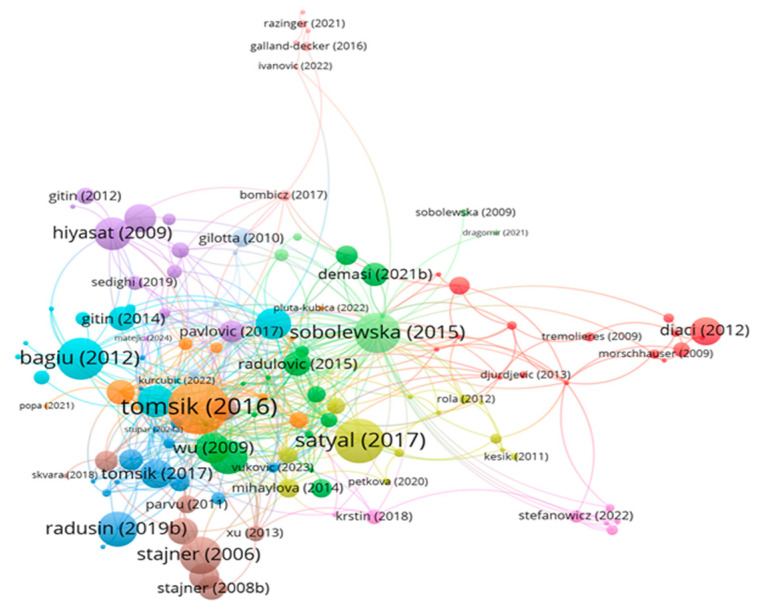
Overview of the most cited authors from the given field of research (2006–2024).

We selected Web of Science as the scientific database to obtain a comprehensive overview of global research results in our reference domain using the VOSviewer software, version 1.6.20. The literature search was conducted in a single stage. The keyword list was designed to capture relevant research on *Allium ursinum* (e.g., wild garlic), food, rural tourism, and gastronomy. This integrated search strategy enabled us to identify academic publications that simultaneously address the gastronomic, cultural, and tourism-related aspects of the plant in rural contexts. These terms were used to generate a comprehensive keyword set for systematic literature searches in the Web of Science database. We identified a sample of 139 manuscripts, retaining only those that were closely related to the topic and articles published in academic journals. Figure 3 provides an overview of the most cited manuscripts by authors from 2006 to 2024, and it can be concluded that a very small number of studies have generally covered the use of wild garlic as an attractor for tourists.

### 2.1. Participants and Setting

**Figure 4:**
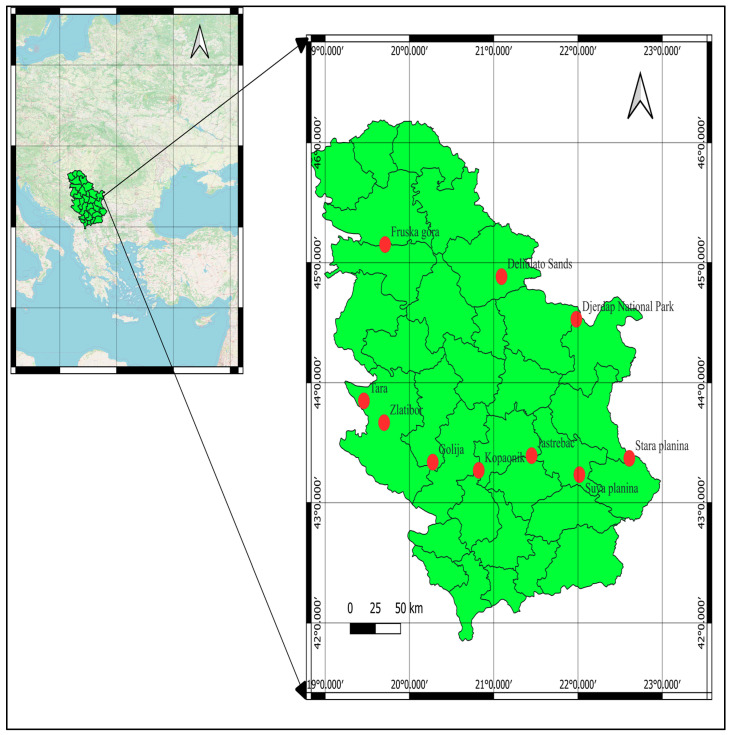
Map of wild garlic harvesting locations in Serbia.

Data collection was conducted from March to May 2024, a specific period when wild garlic (*Allium ursinum*) emerges in the Republic of Serbia. The sample consisted of a total of 336 participants, recruited among tourists who consumed food containing wild garlic or used fresh wild garlic at rural households providing culinary services. A purposive sampling method (judgmental sampling) was applied to recruit participants who had direct experience with consuming *Allium ursinum* products in rural tourism settings. This approach was chosen to ensure that only respondents with relevant exposure to the studied phenomenon were included in the sample [62]. The sample was collected from various rural destinations across Serbia known for the traditional use of *Allium ursinum* in local gastronomy. As illustrated on the map, the study was conducted in selected rural tourism destinations across the Republic of Serbia, specifically in areas known for the seasonal presence of *Allium ursinum* L. and rich gastronomic traditions. Data were collected in the following locations: Fruška Gora, Deliblato Sands, Đerdap National Park, Tara, Zlatibor, Golija, Kopaonik, Jastrebac, Stara Planina, and Suva Planina. These areas were chosen due to their ecological diversity, established rural tourism infrastructure, and cultural relevance in the foraging and preparation of wild edible plants. The geographical distribution of participants included households located near rivers and forested areas, corresponding to the natural habitat of wild garlic (Figure 4).

During data collection, special attention was given to moral hazard and ethical aspects of the research. Participants were informed about the complete anonymity and confidentiality of their responses, as well as the fact that the data were collected exclusively for research purposes. Their participation was entirely voluntary and free from any pressure or potential harmful consequences. The instrument used for data collection was a structured questionnaire with a 5-point Likert scale, where 1 indicated strong disagreement and 5 indicated strong agreement with a given statement. The questionnaire included items related to tourists’ cognitive and affective responses, as well as their revisit intention. Before distributing the main questionnaire, a pilot study was conducted with a sample of 50 participants in February 2024. The aim of the pilot study was to assess the validity, clarity, and reliability of the formulated questions, as well as to identify potential issues in the wording of items or the structure of the questionnaire. Additionally, the pilot study served as an initial test for identifying potential common method bias (CMB) and evaluating the time required to complete the questionnaire. During this process, particular attention was given to involving experts in tourism, gastronomy, and rural development to ensure additional validity of the instruments. Consultations with experts were carried out in two stages. First, the questionnaire was presented to three professors from universities in Serbia who specialize in research on gastronomic tourism and rural development. Their comments focused on the accuracy of question formulation, the adequacy of the scale for measuring affective and cognitive responses, and the relevance of the items in the context of authentic tourism experiences.

The second stage involved consultations with five practitioners from the tourism industry, including managers of rural households providing food services based on local ingredients such as *Allium ursinum*. These consultations enabled the identification of potential shortcomings in question formulation from the perspective of practical application and user experience. Based on the feedback obtained through consultations and the pilot study, necessary changes were made to improve the wording of the items for better understanding. Specifically, certain items were reformulated for greater clarity and simplicity, while additional questions were included to cover extra dimensions of authentic experiences identified by experts as relevant. Moreover, the evaluation scale was revised to ensure that participants’ responses accurately reflect their cognitive and affective responses to the use of *Allium ursinum* in rural tourism.

Table 1 presents the socio-demographic profile of the tourist sample. The majority of participants were female (58.3%) and aged between 30 and 59 years. Most respondents were domestic tourists from Serbia (63.7%), while others came from Germany, Hungary, Austria, Slovenia, and countries such as Italy, the Czech Republic, and Slovakia. Urban residents accounted for 72.1% of the sample. In terms of education, 70.8% held a university degree, and a large portion of respondents reported an average monthly income between EUR 600 and 900. This diversity provides a solid foundation for exploring how demographic characteristics influence perceptions of authentic gastronomic experiences and behavioral intentions in rural tourism settings.

### 2.2. Questionnaire Design

Data were collected using structured questionnaires designed to measure several constructs related to visitor experiences. It is important to note that this research is based on collecting subjective opinions and perceptions of respondents through questionnaires, rather than objective measurement of actual behavioral reactions. The authors designed the questionnaire items based on the constructs defined by the SOR model, drawing on similar studies to ensure relevance and validity. The questionnaire comprised 30 items grouped into eight constructs: authentic experience, cognitive response, affective response, emotional involvement, enjoyment, flow state, attachment to local food, and revisit intention. The measurement of key constructs in this study was conducted using a structured questionnaire with items developed based on relevant literature. The constructs included affective response, cognitive response, emotional involvement, enjoyment, attachment to local food, and revisit intention.

Items for measuring affective and cognitive responses were adapted from studies that apply the SOR model in the context of tourism experiences [46,58]. Items for emotional involvement and enjoyment were developed according to concepts related to experiential theories and tourist satisfaction [41]. Attachment to local food and revisit intention were measured using adapted items from studies on rural and gastronomic tourism [31,55,63]. The questionnaire was prepared in both Serbian and English, considering the diverse background of the tourists visiting rural destinations. The original version was developed in English and then translated into Serbian. To ensure linguistic equivalence, the Serbian version was back-translated into English by an independent bilingual expert who was not involved in the initial translation. Discrepancies were reviewed and resolved through discussion among the research team to ensure conceptual consistency across both versions.

### 2.3. Data Analysis

Descriptive statistics were employed to summarize the main characteristics of the sample and key constructs, including means (m), standard deviations (sd), and reliability coefficients such as Cronbach’s alpha (α). The internal consistency of the constructs was assessed using Cronbach’s alpha, where values above 0.7 indicated good reliability [64]. The study employs different samples for Exploratory Factor Analysis (EFA) and Confirmatory Factor Analysis (CFA) to ensure the validity of the model. The first sample of 168 respondents was used for EFA, while the second sample of 168 respondents was used for CFA to ensure data independence and the reliability of the results. Additionally, Composite Reliability (CR) and Average Variance Extracted (AVE) were calculated to further evaluate the reliability and convergent validity of the constructs [65]. High CR and AVE values confirmed the adequacy of the constructs. To ensure the data’s suitability for factor analysis, the Kaiser–Meyer–Olkin (KMO) measure of sampling adequacy was used, with a KMO value greater than 0.6 indicating good adequacy [65]. Bartlett’s Test of Sphericity was conducted to confirm the appropriateness of the data for factor analysis, with significant results (*p* < 0.001) supporting its suitability [66]. The Kaiser–Meyer–Olkin value of 0.753 indicates moderately good adequacy of the sample, while Bartlett’s test of sphericity shows a statistically significant result (Chi-square (X2) = 5992.092, df = 435, *p* < 0.001). These results confirm that the variables are sufficiently correlated with each other and that factor analysis is appropriate for these data, allowing the identification of latent constructs in the research model. The underlying structure of the components was then identified using exploratory factor analysis (EFA), and the item factor loadings were validated [63].

To identify and control Common Method Bias (CMB), both procedural and statistical techniques were employed. Procedurally, participants were informed about the anonymity and confidentiality of their responses to reduce socially desirable answering. Statistically, Harman’s Single-Factor Test was applied to detect potential bias. The results of the analysis indicated that the first extracted factor accounted for 19.4% of the total variance, which is significantly below the threshold of 50% [67].

Structural model fit (SEM) was assessed using various indices. The standardized root mean square residual (SRMR) was used to assess model fit, with values below 0.08 indicating a good fit [68]. The results of the fit summary analysis show that the model has good fitness indicators. The SRMR values are 0.032 for the saturated model and 0.051 for the estimated model, indicating a good fit. The values of d_ULS (0.028 for the saturated model and 0.050 for the estimated model) and d_G (2.334 for the saturated model and 2.651 for the estimated model) are within acceptable limits. The Chi-square values are low (2.321 for the saturated model and 2.810 for the estimated model), indicating a good model fit [63]. Finally, the NFI values (0.982 for the saturated model and 0.925 for the estimated model) also indicate a satisfactory fit. External variation factor (VIF) values were evaluated to detect multicollinearity between predictor variables (VIF < 3.3). The heterotrait–monotrait (HTMT) ratio was used to assess discriminant validity between constructs (<0.90) [63]. Path analysis was employed to examine the proposed relationships between the constructs. The relationships were assessed by calculating path coefficients, which involved analyzing significance levels (*p*-values), standardized coefficients, and t-values to determine both the strength and direction of these relationships [66]. A moderation analysis was conducted to examine the moderating effects of demographic variables such as age, education, gender, and income on the relationship between affective response and intention to visit. Interaction terms were formed between the affective response and each of the demographic variables to test these effects. The effect of these interaction terms on intention to visit was evaluated to determine whether the strength or direction of the relationship between affective response and intention to visit varied by demographic factors.

## 3. Results

### 3.1. Descriptive Statistics and Factor Analysis of the Construct

The descriptive data analysis revealed that the average scores for most constructs were high, indicating generally positive attitudes among respondents toward the use of *Allium ursinum* in the local gastronomic offering. For example, constructs such as enjoyment (m = 4.75, sd = 0.758) and affective response (m = 4.61, sd = 0.672) showed particularly high average values, suggesting that respondents greatly enjoyed the experience and developed positive emotions during the consumption of food containing *Allium ursinum*. In contrast, constructs such as attachment to local food (m = 3.40, sd = 1.129) and revisit intention (m = 3.73, sd = 1.163) had somewhat lower average scores, which may indicate potential for improving the tourism offering in terms of enhancing visitor loyalty. This result reflects the distinction between affective experience and behavioral intention. As shown by Kim et al. [69], satisfaction can influence the customer’s intention to revisit a restaurant, but this effect is moderated by perceived food healthiness and value. In other words, even if tourists enjoy their meal, they may not intend to return unless additional cognitive or health-related benefits are perceived. This may explain why revisit intention and attachment to local food scored lower than other affective constructs, suggesting that emotional responses alone may not suffice to trigger strong loyalty behaviors. In the initial phase, through the implementation of Exploratory Factor Analysis (EFA), key factors related to authentic experience, cognitive response, enjoyment, affective response, emotional involvement, flow state, attachment to local food, and revisit intention were identified. The factors satisfactorily explained the variance, with Eigenvalue values for all constructs exceeding the recommended threshold of 1.0. These results suggest that the constructs adequately capture the conceptualized tourist experiences with *Allium ursinum*. The percentage of explained variance for each construct was sufficiently high to justify its retention for further analysis.

In the subsequent phase, Confirmatory Factor Analysis (CFA) confirmed the validity of the identified constructs through the assessment of internal consistency, convergent, and discriminant validity. All constructs demonstrated high internal consistency, with Cronbach’s alpha (α) values exceeding the recommended threshold of 0.70. Furthermore, high values of Composite Reliability (CR) and Average Variance Extracted (AVE) indicate satisfactory convergent validity for all constructs. The composite reliability of all constructs exceeds the threshold of 0.70, while AVE values are generally above the recommended threshold of 0.50, indicating that the constructs successfully capture the variance of their items. The identified constructs related to authentic experience, cognitive and affective response, emotional involvement, enjoyment, flow state, attachment to local food, and revisit intention were confirmed as valid and reliable within the proposed model. The results indicate that *Allium ursinum* is perceived as an authentic local ingredient that contributes to the creation of unique experiences, which in turn reflects positive cognitive and emotional reactions among tourists. Constructs such as enjoyment and emotional involvement demonstrated a high degree of internal consistency, indicating that specific experiences with *Allium ursinum* in local gastronomy are coherent and consistently perceived among respondents. This further supports the notion that the use of indigenous plants in local cuisine can serve as a significant stimulus for creating authentic tourist experiences. Ultimately, the validation of the identified constructs through CFA suggests that the model is well-fitted and adequately encompasses aspects of the tourist experience with *Allium ursinum*. The findings confirm that authentic experience and cognitive and affective responses, as well as attachment to local food, are relevant indicators of revisit intention, which aligns with the theoretical framework of the SOR model (Table 2).

### 3.2. Results of the SEM Analysis

The results of the SEM analysis (structural equation modeling) show different values of R2 (R-squared). The affective response can be explained by the model in 25.4% of the cases, which means that about a quarter of the variation in this response is predicted by the model. Local food approval has an even higher percentage: 40.3% of the variance is explained by the model, indicating a strong relationship between the model and this factor. Cognitive response was explained in 37.2% of cases, which confirms the significant influence of the model on respondents’ thinking. Emotional engagement, on the other hand, explained 24.3% of the variance, while fun accounted for a lower percentage of 19.7%. A similar trend can be observed for the flow state, where the model explained 20.7% of the variance. The intention to revisit shows a high percentage of explained variance of 40.3%, indicating that the model contributes significantly to understanding respondents’ intention to revisit the destination. The VIF (variance inflation factor) values for different variables in the model. VIF values were used to assess multicollinearity among variables. Most variables had VIF values below 3, suggesting no serious multicollinearity [65].

The results show a high reliability and validity of the constructs (Table 3). Most constructs have a Cronbach’s alpha (α) value of over 0.8, indicating high internal consistency. The composite reliability (CR) and the average variance extracted (AVE) also show high values, which further confirms the validity of the constructs. Taken together, these indicators suggest that the measurements are reliable and valid for further analysis.

**Figure 5:**
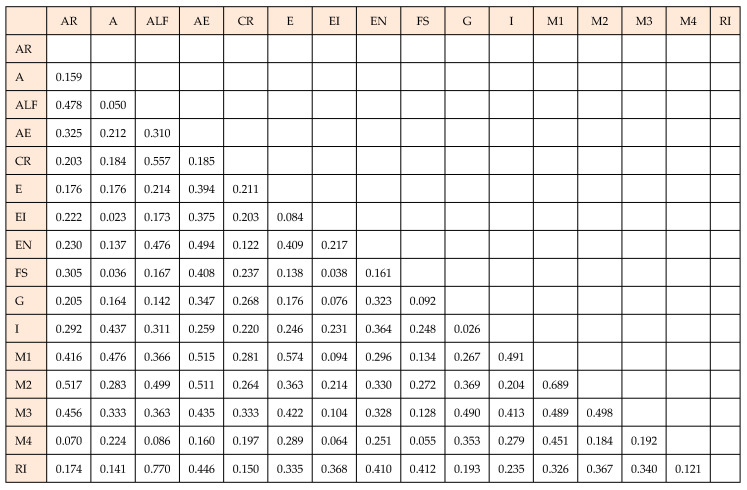
Heterotrait–monotrait ratio. Note: AR—affective response, A—age, ALF—attachment to local food, AE—authentic experience, CR—cognitive response, E—education, EI—emotional involvement, EN—enjoyment, FS—flow state, G—gender, I—income, M1–M4—moderation effects.

The results of the heterotrait–monotrait ratio (HTMT) analysis show that all constructs have values below 0.9, which indicates adequate discriminative validity [63]. These findings confirm that the constructs are mutually different and that each one measures different aspects of the investigated phenomena (Figure 5).

The results shown in Table 4 of the model selection criteria show different values of the criteria, such as AIC, AICu, AICc, BIC, HQ, and HQc, for each construct. Low values of these criteria indicate a better fit of the model. Based on the results in Table 3, it is concluded that the affective response and cognitive response are the best models, with the lowest AIC and BIC values, indicating their superior fit. The revisit intention shows a weaker model fit with higher AIC and BIC values. Other constructs have a moderate model fit. Therefore, the affective and cognitive responses best explain the variances in the data compared to the other constructs.

The results of the path analysis indicate a statistically significant effect of affective response on attachment to local food (β = 0.356, *p* = 0.000), confirming the importance of emotional reactions in shaping positive relationships with local ingredients (H3d). This association is further supported by the significant influence of affective response on emotional involvement (H3a; β = 0.154, *p* = 0.030), enjoyment (H3b; β = 0.312, *p* = 0.005), and flow state (H3c; β = 0.141, *p* = 0.032). The presence of positive emotions during authentic experiences significantly contributes to the formation of specific affective states, which supports the theoretical foundations of the SOR model. Additionally, affective response shows a statistically significant direct effect on revisit intention (H3e; β = 0.242, *p* = 0.023), indicating that positive emotions generated during interaction with local ingredients can effectively contribute to tourists’ long-term loyalty toward the destination. Furthermore, attachment to local food significantly influences revisit intention (H4; β = 0.353, *p* = 0.027), which corroborates previous research on the importance of affective connections in generating behavioral intentions. The findings suggest that authentic experience significantly influences affective response (H1a; β = 0.310, *p* = 0.002) and cognitive response (H1b; β = 0.610, *p* = 0.000), indicating that experiences involving local ingredients such as *Allium ursinum* not only elicit emotional reactions but also contribute to acquiring knowledge and understanding the cultural context. Furthermore, cognitive response significantly affects affective response (H2a; β = 0.335, *p* = 0.000), supporting the assumption that cognitive processes precede affective reactions, consistent with the SOR model. Moreover, cognitive response has a significant impact on attachment to local food (H2c; β = 0.264, *p* = 0.000) and revisit intention (H2b; β = 0.427, *p* = 0.002), suggesting that perceptions of authenticity and knowledge acquisition directly contribute to tourists’ long-term loyalty. These findings confirm the importance of integrating cognitive and affective responses in analyzing behavioral intentions, particularly in the context of authentic gastronomic experiences. The moderating effects of age (M4), education (M3), gender (M1), and income (M2) on the relationship between affective response and revisit intention were not statistically significant (H5a; β = 0.040, *p* = 0.433; H5b; β = −0.023, *p* = 0.821; H5c; β = 0.004, *p* = 0.979; H5d; β = 0.087, *p* = 0.231) (Table 5).

**Figure 6:**
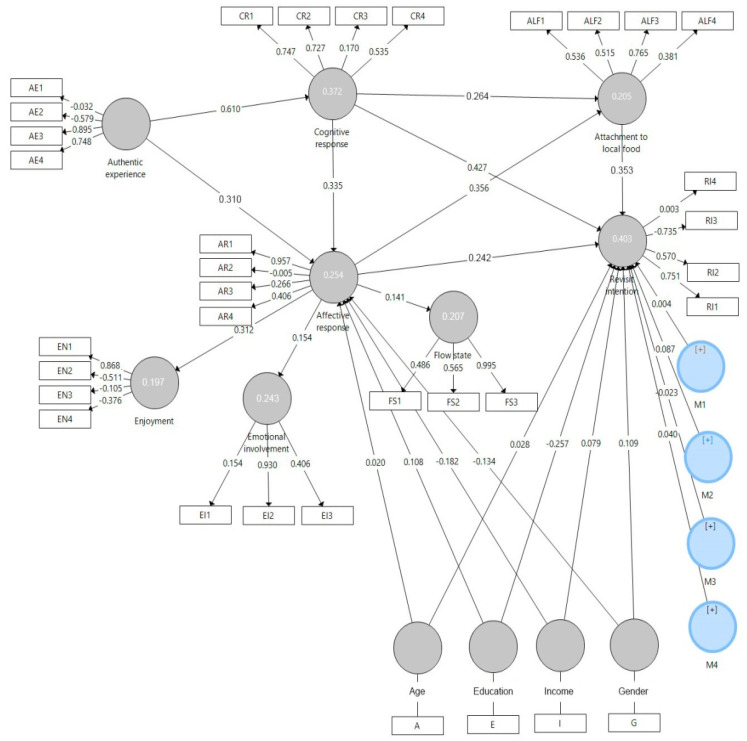
A structural model with paths and latent research variables.

Figure 6 shows the structural model with the paths connecting the different constructs and latent variables of the study. The model shows the relationships between constructs such as authentic experience, affective response, cognitive response, enjoyment, emotional involvement, flow state, commitment to local food, and intention to visit. The model also includes moderating variables such as age, education, income, and gender. The figure shows the path coefficients, standard errors, and R^2^ values for each construct, providing a detailed overview of the influence and significance of each relationship in the model.

## 4. Discussion

The results of this study confirm the importance of authentic experiences in rural gastronomic tourism, which clearly aligns with the existing literature emphasizing authenticity as a key element in differentiating tourism offerings. Previous research indicates that the use of local ingredients can significantly contribute to creating unique tourist experiences, particularly in rural areas where cultural heritage and tradition play a vital role [26,27]. Such an approach allows tourists to establish a deeper connection with the destination by experiencing the originality and uniqueness of local cuisine. The connection between authentic experiences and positive cognitive and affective responses is further highlighted by the findings of Dimitrijević et al. [40] and Blešić et al. [34], who emphasized that experiences shaped by authentic elements, such as specific ingredients, not only enhance perceptions of authenticity but also contribute to knowledge acquisition and understanding of the cultural context. This process of acquiring knowledge is reflected in positive affective reactions, which further supports the SOR model, where cognitive responses precede affective reactions.

Accordingly, the findings of this study suggest that perceptual and cognitive processes largely shape tourists’ emotional responses, thus confirming Mehrabian and Russell’s [46] assertions about the relationship between internal reactions and behavioral responses. These insights indicate that cognitive responses arising from authentic experiences, such as perceptions of authenticity and acquiring new knowledge, play a crucial role in generating positive emotions such as enjoyment, emotional involvement, and flow state. This is consistent with the findings of Lin and Hsu [51], who emphasized that authentic experiences not only enhance perceptions of authenticity but also support sustainable development by promoting cultural awareness and valuing local resources. In this context, emotional responses are not merely transient experiences but serve as a foundation for deeper attachment to the destination, which is essential for fostering tourist loyalty. The connection between positive affective reactions and behavioral intentions is further supported by the research of Jokom et al. [55], which shows that enjoyment and emotional involvement can significantly contribute to revisit intention and destination recommendation. In this study, emotional involvement, enjoyment, and flow state are highlighted as key affective responses that transfer to behavioral intentions. When tourists experience a high level of emotional satisfaction and involvement, it is more likely that positive intentions, such as loyalty to the destination and repeat visits, will develop.

These findings are further strengthened by the research of Dai et al. [56], who emphasized the importance of positive affective responses in strengthening attachment to local products, thereby increasing the likelihood of repeat visits. This study provides similar conclusions, confirming that affective responses, such as enjoyment, emotional involvement, and flow state, are critical components in shaping tourists’ behavioral intentions. Such emotional connections with local products play a crucial role in creating long-term loyalty, which is particularly important for destinations seeking to differentiate themselves through authentic gastronomic experiences.

Furthermore, these results indicate that positive cognitive responses are not only manifested through acquiring knowledge and understanding the cultural context but also through forming emotional connections with the destination. This is consistent with the findings of Xu et al. [60], who emphasized that emotional involvement can enhance attachment to specific cultural products and contribute to the sustainable development of rural destinations. These results suggest that emotional responses derived from authentic experiences have the potential to contribute not only to tourist satisfaction but also to long-term commitment to the destination. The findings of this study provide a strong empirical basis for asserting that authentic gastronomic experiences significantly influence tourists’ cognitive and affective responses, which further reflect on behavioral intentions such as revisit intention and loyalty to the destination. Such findings offer valuable insights for improving tourism offerings in rural destinations through the promotion of authenticity and the use of specific local ingredients such as *Allium ursinum*. This section may be divided by subheadings. It should provide a concise and precise description of the experimental results, their interpretation, as well as the experimental conclusions that can be drawn.

## 5. Conclusions

The obtained results proved a basis for understanding and evaluating the importance of wild garlic as a factor in enhancing the tourism offer in rural areas. By utilizing this unique natural resource, rural communities can enrich their gastronomic offer and create authentic experiences that can be crucial for attracting and retaining tourists. This research confirms that certain local ingredients can play an important role in the cultural and economic development of rural areas by promoting sustainability and cultural preservation. In response to the research question posed in the introduction, the findings of this study confirm that authentic gastronomic experiences involving *Allium ursinum* significantly influence both cognitive and affective responses among tourists. These internal responses, in turn, shape behavioral intentions such as attachment to local food and revisit intention, thereby validating the theoretical assumptions of the SOR model in a rural tourism context.

### 5.1. Theoretical Implications

This paper provides a novel theoretical contribution by applying the SOR model in the specific context of rural gastronomic tourism, expanding the understanding of the impact of local ingredients such as *Allium ursinum* on tourists’ perceptions, emotional reactions, and behavioral intentions. The innovativeness of this study lies in integrating cognitive, affective, and behavioral responses in the analysis of authentic experiences, which previous research has rarely considered as a comprehensive structure. A particular contribution is reflected in the identification of specific components of the affective response (enjoyment, emotional involvement, and flow state) that arise from authentic gastronomic experiences. These findings are built upon the works of Jokom et al. [55] and Xu et al. [60], who highlighted the importance of positive emotional reactions in creating attachment to local products and revisit intentions. The study also advances theoretical knowledge about the role of cognitive responses in shaping emotional reactions, which has been identified as a critical aspect in understanding tourist behavior. The new insights provided by this research relate to the broader application of the SOR model in analyzing the impact of authentic experiences in tourism. By connecting cognitive, affective, and behavioral responses, this paper enhances the theoretical understanding of the complexity of tourist experiences in rural settings. Moreover, the integration of specific local ingredients such as *Allium ursinum* offers an innovative theoretical framework that can be applicable to other local products across various cultural contexts. Although the findings align with previous research, it should be noted that they are based on subjective assessments of tourists’ perceptions, which may present a limitation in terms of objectively measuring the impact of authentic experiences. The subjective nature of perception makes it challenging to clearly identify the actual influence of *Allium ursinum* on tourists’ behavioral intentions, which opens space for further research involving quantitative methods or comparative studies across different geographical and cultural contexts [70].

### 5.2. Practical Implications

The findings of this study have significant practical implications for enhancing rural tourism through the integration of wild garlic (*Allium ursinum*) into the tourism offer. Utilizing *Allium ursinum* as a key ingredient for developing authentic gastronomic products can significantly contribute to attracting tourists seeking unique and authentic experiences while promoting their connection to local culture and traditions. Integrating specific local ingredients into the tourism offer can be achieved through organizing specialized gastronomic tours [71,72,73], culinary workshops, and tastings based on the use of wild garlic. Such activities not only contribute to the differentiation of the tourism offer but also to the creation of positive emotions among tourists, which can further influence their intention to revisit. Moreover, promoting local gastronomic experiences involving *Allium ursinum* can enhance the attractiveness of destinations and motivate tourists to return to rural households. The direct sale of *Allium ursinum* products at local markets, fairs, and tourism events can further contribute to the economic development of rural communities, increasing household income and providing opportunities for improving tourism infrastructure. Developing specialized tourism products based on *Allium ursinum* can be considered an effective way of preserving and promoting cultural heritage, which is particularly important in the context of rural tourism. By connecting authentic gastronomic experiences with local culture and traditions, rural destinations can create a long-term sustainable tourism offer that stands out from competing destinations. Furthermore, incorporating *Allium ursinum* into the tourism offer provides opportunities for creating seasonal events that could attract specific target groups of tourists, particularly those interested in gastronomy and natural products. Additionally, the strategic promotion of *Allium ursinum* products through digital platforms, social networks, and tourism guides can significantly enhance the visibility of destinations that include it in their offer. The application of innovative marketing approaches, such as collaborative projects between local communities, tourism agencies, and institutions, can further improve the promotion and distribution of *Allium ursinum* products. In this way, rural tourism can become more attractive to a broader range of visitors while simultaneously providing economic, social, and cultural benefits for local communities.

### 5.3. Limitations and Future Research Directions

Although the findings of this study are promising, there are certain limitations that must be considered to accurately interpret the results and identify opportunities for future research. The study was conducted on a limited sample of participants from a specific geographical area, which may affect the generalizability of the results. The focus on rural destinations in the Republic of Serbia implies that the findings cannot be directly applied to other countries or regions with different cultural and gastronomic traditions. Various regions may have specific ways of preparing and consuming *Allium ursinum*, further complicating comparisons with findings from other geographical contexts. Another limitation concerns the timeframe within which data collection was conducted. Data were collected from March to May 2024, which corresponds to the natural growth season of *Allium ursinum* in the Republic of Serbia. This seasonal approach may significantly influence the results, as *Allium ursinum* is available in fresh form only during this limited period. Additionally, the seasonality of the plant itself affects the possibility of continuous research, as tourists visiting destinations outside this period cannot have the same experience with *Allium ursinum*-based products. Such limitations may negatively impact the ability to draw long-term conclusions about tourist behavior and intentions, particularly concerning revisit intention. Further challenges arise from the fact that not all rural households and restaurants include *Allium ursinum* in their offerings. This could result in a limited sample of respondents who have had direct experiences with *Allium ursinum* products, potentially affecting the accuracy and reliability of the findings. Moreover, many tourists are unfamiliar with the use or existence of *Allium ursinum* as a specific local ingredient, which may adversely affect their ability to adequately evaluate their gastronomic experience. This limitation is particularly pronounced among tourists from urban areas or regions where *Allium ursinum* is not traditionally used in local cuisine.

Besides these methodological limitations, there is also the issue of cultural and culinary heritage, which was not sufficiently considered. The use of *Allium ursinum* may be valued differently across cultural contexts, which could affect perceptions of authenticity and overall tourist satisfaction. Additionally, limitations related to sampling and the data collection timeframe may hinder comparisons with other studies dealing with similar topics in different cultural and geographical contexts. To overcome these limitations, future research should include a broader geographical area and a more diverse sample of participants. Repeated studies during different seasons are also recommended to provide a more comprehensive picture of tourist behavior and intentions. Comparing findings with studies from other countries or regions could offer deeper insights into the universality or specificity of *Allium ursinum’s* impact on tourist experiences and revisit intentions. Although the results of this study generally align with previous findings, their interpretation must be approached with caution due to the inherent limitations arising from the subjective nature of perception assessment. These findings provide valuable insights into the relationship between authentic experiences and tourists’ behavioral intentions, while also highlighting the need for further research to more precisely clarify the nature of these connections. Another limitation concerns the potential for respondent bias, as participants were aware of the purpose of the study and the specific focus on *Allium ursinum.* This awareness may have influenced their responses, especially in self-reported affective and behavioral intentions, possibly leading to overstatement due to social desirability. Future research could address this limitation by using more indirect or implicit measurement techniques or by implementing blind experimental designs to minimize expectancy effects. A further limitation of the study is that all survey items used in the measurement model were positively worded. While this was intended to enhance clarity and consistency in participant interpretation, it may have increased the risk of response bias, particularly acquiescence bias. Future studies could benefit from incorporating a mix of positively and negatively worded items to improve scale balance and minimize such bias. Some of the items used to assess flow state and affective responses were formulated with relatively strong emotional language, which may not reflect the typical experience of all tourists. This could have led to lower endorsement or reduced sensitivity in capturing moderate levels of engagement. Future studies may consider using more neutral or graduated phrasing to better capture varying degrees of emotional involvement.

Given the limitations of the present study, future research should aim to broaden the geographical scope and include diverse participant samples to enhance the generalizability of findings. Comparative studies across different regions and cultures could provide deeper insights into the universality or specificity of the impact of *Allium ursinum* on tourist experiences and revisit intentions. Longitudinal studies are also recommended to examine changes in tourist perceptions and behaviors over extended periods, especially considering the seasonal nature of *Allium ursinum*. Additionally, future research could integrate quantitative measures of actual tourist behaviors, not just perceptions, to establish a clearer causal relationship between cognitive and affective responses and behavioral intentions. Incorporating mixed-method approaches or experimental designs could further enhance the robustness and reliability of findings. Finally, exploring the interplay between cultural heritage, culinary tourism, and sustainability in different geographical and cultural contexts could provide valuable insights for enhancing the theoretical framework and practical applications of this research.

## Figures and Tables

**Table 1:** Socio-demographic characteristics of the sample.

Characteristics	Category	Frequency (%)
Gender	Male	140 (41.7%)
	Female	196 (58.3%)
Age	18–29 years	82 (24.4%)
	30–44 years	128 (38.1%)
	45–59 years	92 (27.4%)
	60+ years	34 (10.1%)
Country of origin	Serbia	214 (63.7%)
	Germany	42 (12.5%)
	Hungary	28 (8.3%)
	Austria	18 (5.4%)
	Slovenia	14 (4.2%)
	Others (Italy, Czech Republic, Slovakia)	20 (5.9%)
Place of residence	Urban	242 (72.1%)
	Rural	94 (27.9%)
Education	Secondary education	98 (29.2%)
	Higher education (BA/MA/PhD)	238 (70.8%)
Monthly income	Below average (EUR <600)	116 (34.5%)
	Average (EUR 600–900)	146 (43.5%)
	Above average (EUR >900)	74 (22.0%)

**Table 2:** Descriptive statistics and reliability of constructs.

Factor	Statement	m	sd	α	FA
Authentic experience	*Allium ursinum* in local food provided me with authentic experiences.	3.76	1.054	0.647	0.884
m = 3.88, sd = 1.131 α = 0.992 CR = 0.894, AVE = 0.678 Eigenvalues—5.453 Variance explained—18.176	*Allium ursinum* in local food provided me with genuine experiences.	4.18	0.887	0.653	0.830
	*Allium ursinum* in local food provided me with exceptional experiences.	3.69	1.380	0.666	0.827
	*Allium ursinum* in local food provided me with unique experiences.	3.90	1.203	0.670	0.746
Cognitive response	*Allium ursinum* in local food helps me gain knowledge about local traditions.	3.90	0.976	0.687	0.825
m = 4.28, sd = 0.911 α = 0.960 CR = 0.861, AVE = 0.610 Eigenvalues—3.644 Variance explained—12.147	*Allium ursinum* in local food is beneficial for my health.	4.19	1.140	0.699	0.823
	*Allium ursinum* in local food is useful for expanding culinary knowledge.	4.57	0.725	0.701	0.794
	*Allium ursinum* in local food allows me to form friendships with other food enthusiasts.	4.49	0.805	0.710	0.669
Enjoyment	*Allium ursinum* in local food is enjoyable for me.	4.66	0.650	0.720	0.803
m = 4.75, sd = 0.758 α = 0.955 CR = 0.865, AVE = 0.617 Eigenvalues—2.106 Variance explained—7.020	*Allium ursinum* in local food provides me with satisfaction.	4.43	0.975	0.735	0.799
	*Allium ursinum* in local food is fun for me.	4.66	0.636	0.747	0.795
	*Allium ursinum* in local food makes me happy.	4.55	0.732	0.758	0.744
Affective response	*Allium ursinum* in local food evokes positive emotions in me.	4.22	1.221	0.763	0.629
m = 4.61, sd = 0.672 α = 0.800 CR = 0.837, AVE = 0.564 Eigenvalues—1.686 Variance explained—5.619	*Allium ursinum* in local food makes me feel happy and satisfied.	4.88	0.322	0.770	0.833
	*Allium ursinum* in local food makes me emotionally attached to local customs.	4.54	0.730	0.780	0.781
	*Allium ursinum* in local food provides me with a sense of connection to the culture.	4.83	0.417	0.799	0.744
Emotional involvement	I am fully engaged in the experience of using *Allium ursinum* in local food.	4.36	1.033	0.800	0.700
m = 4.32; sd = 1.028 α = 0.987 CR = 0.855, AVE = 0.663 Eigenvalues—1.584 Variance explained—5.281	I am deeply impressed by the use of *Allium ursinum* in local food.	4.30	1.024	0.810	0.680
	I feel complete empathy towards experiences related to *Allium ursinum* in local food.	4.30	1.027	0.822	0.720
Flow state	When enjoying local food with *Allium ursinum*, I feel completely absorbed.	4.24	1.038	0.836	0.710
m = 4.27, sd = 1.033 α = 0.971 CR = 0.802, AVE = 0.577 Eigenvalues—1.407 Variance explained—4.689	When enjoying local food with *Allium ursinum*, time flies by quickly.	4.27	1.029	0.845	0.690
	When enjoying local food with *Allium ursinum*, I forget all my worries.	4.30	1.024	0.850	0.680
Attachment to Local Food	I am closely connected to the experiences of using *Allium ursinum* in local food.	4.28	1.028	0.869	0.750
m = 3.40, sd = 1.129 α = 0.901 CR = 0.839, AVE = 0.569 Eigenvalues—1.285 Variance explained—4.283	Using *Allium ursinum* in local food is part of my life.	4.22	1.042	0.874	0.770
	I am attached to the use of *Allium ursinum* in local food.	2.78	1.068	0.880	0.760
	Using *Allium ursinum* in local food is important to me.	2.34	1.378	0.895	0.780
Revisit intention	I want to explore destinations where *Allium ursinum* is used in local cuisine.	3.90	1.315	0.901	0.800
m = 3.73, sd = 1.163 α = 0.875 CR = 0.872, AVE = 0.632 Eigenvalues—1.174 Variance explained—3.914	I am attracted to places where I can try dishes with *Allium ursinum*.	2.93	1.112	0.983	0.820
	I plan to visit restaurants that offer specialties with *Allium ursinum*.	3.60	1.463	0.924	0.840
	I am interested in visiting destinations where *Allium ursinum* is a key ingredient in dishes.	4.49	0.764	0.930	0.860

Note: m—arithmetic mean, sd—standard deviation, α—Cronbach alpha, FA—factor loading, CR—composite reliability, AVE—average variance extracted.

**Table 3:** Construct reliability and validity.

	α	rho_A	CR	AVE
Affective response	0.976	0.804	0.880	0.688
Age	0.080	0.822	0.900	0.600
Attachment to local food	0.868	0.824	0.840	0.621
Authentic experience	0.983	0.849	0.816	0.624
Cognitive response	0.916	0.842	0.846	0.651
Education	0.920	0.847	0.903	0.674
Emotional involvement	0.895	0.897	0.833	0.551
Enjoyment	0.810	0.863	0.935	0.592
Flow state	0.879	0.875	0.842	0.615
Gender	0.917	0.833	0.817	0.599
Income	0.945	0.852	0.919	0.618
M1	0.874	0.800	0.905	0.644
M2	0.866	0.894	0.855	0.629
M3	0.769	0.888	0.830	0.607
M4	0.745	0.805	0.955	0.668
Revisit intention	0.830	0.868	0.819	0.658

Note: α—Cronbach’s alpha, CR—composite reliability, AVE—average variance extracted, M1–M4—moderation effects.

**Table 4:** Criteria for model selection.

Constructs	AIC	AICu	AICc	BIC	HQ	HQc
Affective response	−131.797	−124.747	−110.200	−102.380	−120.248	−119.774
Attachment to local food	−108.629	−105.620	−96.500	−96.022	−103.679	−103.567
Cognitive response	−227.159	−225.155	−218.500	−218.754	−223.860	−223.800
Emotional involvement	−27.823	−25.819	−20.500	−18.418	−25.523	−25.463
Enjoyment	−47.609	−45.605	−40.500	−39.204	−44.309	−44.249
Flow state	−26.908	−24.904	−23.500	−21.497	−23.609	−23.549
Revisit intention	−231.860	−219.712	−210.500	−181.429	−212.061	−210.783

Note: AIC—Akaike’s information criterion, AICu—unbiased Akaike’s information criterion, AICc—corrected Akaike’s information criterion, BIC—Bayesian information criterion, HQ—Hannan–Quinn criterion, HQc—corrected Hannan–Quinn criterion.

**Table 5:** Path coefficients and hypothesis testing.

Path	Effect	m	sd	t	*p*	Confirmation	
Affective response → Attachment to local food	0.356	0.356	0.045	7.930	0.000	H3d	✔
Affective response → Emotional involvement	0.154	0.076	0.148	1.038	0.030	H3a	✔
Affective response → Enjoyment	0.312	0.223	0.240	1.298	0.005	H3b	✔
Affective response → Flow state	0.141	0.112	0.118	1.197	0.032	H3c	✔
Affective response → Revisit intention	0.242	0.137	0.070	2.033	0.023	H3e	✔
Attachment to local food → Revisit intention	0.353	0.152	0.069	2.218	0.027	H4	✔
Authentic experience → Affective response	0.310	0.164	0.053	3.055	0.002	H1a	✔
Authentic experience → Cognitive response	0.610	0.612	0.028	21.756	0.000	H1b	✔
Cognitive response → Affective response	0.335	0.334	0.061	5.534	0.000	H2a	✔
Cognitive response → Attachment to local food	0.264	0.168	0.040	4.086	0.000	H2c	✔
Cognitive response → Revisit intention	0.427	0.405	0.138	3.091	0.002	H2b	✔
Affective response → (M1) → Revisit intention	0.004	−0.002	0.154	0.026	0.979	H5c	X
Affective response → (M2) → Revisit intention	0.087	0.077	0.073	1.199	0.231	H5d	X
Affective response → (M3) → Revisit intention	−0.023	−0.019	0.101	0.226	0.821	H5b	X
Affective response → (M4) → Revisit intention	0.040	0.040	0.051	0.785	0.433	H5a	X

## Data Availability

The data presented in this study are available on request from the corresponding author.
